# Sampling and Analysis of Odorants and Volatile Organic Compounds in Air, Water, and Soil

**DOI:** 10.1155/2012/308013

**Published:** 2012-12-05

**Authors:** Ki-Hyun Kim, David Parker, Fusheng Li, Kea-Tiong Tang

**Affiliations:** ^1^Atmospheric Environment Laboratory, Department of Environment and Energy, Sejong University, Goon Ja Dong 98, Seoul 143-747, Republic of Korea; ^2^Palo Duro Research Center, West Texas A&M University, Canyon, TX 79016, USA; ^3^College of Agriculture, Guangxi University, Nanning, Guangxi 530005, China; ^4^Department of Electrical Engineering, National Tsing Hua University, No. 101, Sec. 2, Kuang-Fu Road, Hsinchu 30013, Taiwan

As the environmental significance of odor pollution is recognized by the public, the demand for accurate assessment of these pollutants has been growing steadily. Hence, the analytical methodologies commonly used for their determination need to be evaluated from various respects. This special issue is thus intended to compile up-to-date knowledge on the determination of odor pollution with emphasis on volatile organic compounds (VOCs) and many key odor components such as reduced sulfur compounds (RSCs), organic acids, carbonyls, and nitrogenous compounds (ammonia and trimethyl amine).

The publication of this special issue has been achieved by the warm support and devotion of the contributing authors as well as the important contribution offered by expert reviewers. This special issue is intended to provide a snapshot in the timeline of international research and policy of odorant and VOC pollution and to bring together some of the recent and most exciting developments in the area, and to describe the main trends of their application in the field study. It contains a mixture of papers describing the latest development for their application relevant to air pollution and odor pollution.

*Ki-Hyun Kim* *Ki-Hyun Kim* *David Parker* *David Parker* *Fusheng Li* *Fusheng Li* *Kea-Tiong Tang* *Kea-Tiong Tang*

